# Children as frequent attenders in primary care: a systematic review

**DOI:** 10.3399/bjgpopen20X101076

**Published:** 2020-09-02

**Authors:** Mohammed N Al-Saffar, Benedict WJ Hayhoe, Matthew J Harris, Azeem Majeed, Geva Greenfield

**Affiliations:** 1 Department of Primary Care and Public Health, School of Public Health, Imperial College London, London, UK

**Keywords:** Children, Mental Health, Frequent Attenders, Characteristics, Primary Health Care, Chronic Disease, Anxiety, Healthcare Utilisation

## Abstract

**Background:**

Frequent paediatric attendances make up a large proportion of a GP's workload. Currently, there is no systematic review on frequent paediatric attendances in primary care.

**Aim:**

To identify the sociodemographic and clinical characteristics of children who attend primary care frequently.

**Design & setting:**

A systematic review.

**Method:**

The electronic databases MEDLINE, Embase, and PsycINFO were searched up to January 2020, using terms relating to frequent attendance in primary care settings. Studies were eligible if they considered children frequently attending in primary care (aged 0–19 years). Relevant data were extracted and analysed by narrative synthesis.

**Results:**

Six studies, of fair quality overall, were included in the review. Frequent attendance was associated with presence of psychosocial and mental health problems, younger age, school absence, presence of chronic conditions, and high level of anxiety in their parents.

**Conclusion:**

Various sociodemographic and medical characteristics of children were associated with frequent attendance in primary care. Research on interventions needs to account for the social context and community characteristics. Integrating GP services with mental health and social care could potentially provide a response to medical and psychosocial needs of frequently attending children and their families.

## How this fits in

Systematic review literature shows that adult frequent attenders make up a large proportion of the GP workload, but no previous systematic review on children frequent attenders exists. This review indicates that child frequent attenders have differing medical, psychological, and social characteristics. Future strategies need to align GP services with social care to address the psychosocial aspect to improve child health and reduce attendance among frequent attending children.

## Introduction

Primary care services in the UK are under pressure due to increasing patient demand and growing case complexity. Such demand might be intensified by repeat visits by the same patients. Frequent attenders are a small group of primary care patients who attend for a disproportionate amount of primary care visits.^1^ Consequently, health services providers, whether public or private, are interested in providing appropriate solutions for patients who make up a large proportion of GP workload.^2^ A previous review on frequent attenders reported that the top 10% of attenders accounted for 30% to 50% of all primary care consultations.^3^ Frequent attendance in primary care is a recurrent behaviour, where 40% of users are likely to continue to frequently attend in the following year of follow-up.^3^ Frequent attenders in primary care tend to have more chronic physical illnesses than non-frequent attenders,^4^ as well as mental health conditions,^5^ psychological issues,^6^ poor health beliefs,^7^ and are more likely to use other healthcare services.^8^ Despite their obvious health needs, they may be receiving poor care at primary care level.^9^

Older people with long-term conditions will naturally tend to visit their GP frequently, however frequent visits of children may indicate underlying unmet medical or psychosocial needs.^10^ In the UK, clinical workload in primary care has increased by 9.3% between 2007 and 2014 among children below 5 years of age,^11^ however, the reasons for this increase are unclear. Previous reviews on frequent attendance in primary care have focused on adults.^3,12^ A better understanding of the characteristics of these frequently attending children will better facilitate targeted approaches to meet their health needs, and reduce the need for frequent primary care visits. Hence, this study aims to identify characteristics associated with frequent attendance of primary care services by children.

## Method

A systematic review was conducted according to the Preferred Reporting Items for Systematic Reviews and Meta-Analysis guidelines (see Supplementary Table 3).

### Eligibility criteria

Studies on frequent attendance in primary care were included if they focused on children aged 0–19 years according to the World Health Organization definition,^13^ and included data on associated patient characteristics, such as sociodemographics, medical conditions, or clinical characteristics.

As there is no clear definition for frequent attenders, no fixed definition was applied, and definitions were extracted as reported in individual studies. Observational or randomised controlled trial studies, and studies carried out in primary care published in English after 1965 were included. Studies with no quantitative data, such as qualitative studies, were excluded as the study focused on objective measures. No hand searches were performed. The search was updated, up to January 2020.

### Information sources

The electronic databases MEDLINE (Ovid Interface, 1948 onwards), Embase (Ovid Interface, 1980 onwards), and PsycINFO (Ovid Interface, 1806–2018) were searched for relevant articles. The search strategy is detailed in Supplementary Appendix S1.

### Screening and selection

After deduplication, two authors (MA and BH) screened all titles and abstracts identified from searches, before full text screening against eligibility criteria. Discrepancies were resolved by consensus with a third author (GG).

### Data extraction

Data relevant to the study question were extracted from included studies and summarised. Data included sociodemographic and clinical characteristics associated with frequent attendance, and data on frequent attendance rates among children attending primary care clinics. MA, BH, and GG developed the extraction tool (Supplementary Table 1). Data extraction was performed by MA, and verified by BH and GG. Data extracted were characteristics or factors such as demographic, socioeconomic status, and medical characteristics associated with the frequent attendance. Extracted data included author, study design, frequently attendance definition, sample, and key outcomes.

### Quality assessment

After full text assessment of relevant articles, studies were critically appraised for risk of bias by three authors (MA, BH, and GG) using the National Heart, Lung, and Blood Institute quality assessment tool for observational and cross-sectional studies. Studies were not excluded based on quality assessment.

### Outcome measures

The main outcome measure was frequent attendance in primary care. As frequent attendances were defined differently in each study, this review included any definition used by the studies.

### Data synthesis

Due to the limited number of studies identified, as well as heterogeneity of outcomes considered, meta-analysis could not be performed. A narrative synthesis of the data was therefore carried out.

## Results

### ​Characteristics of included studies

Searches identified 2966 articles, of which six met the eligibility criteria (Supplementary Figure 1). Table 1 summarises the characteristics of the included studies. Studies were conducted between 1987 and 2019, and participants were between 0–19 years of age. Three studies were from the UK,^14–16^ one from Croatia,^17^ one from Spain,^18^ and one from the USA.^19^ All studies were observational in design; three were cross-sectional,^14,15,18^ two were prospective cohort,^16,19^ and one a retrospective cohort.^17^

**Table 1.** Study characteristics of studies meeting eligibility criteria for data synthesis

**Author(s**)	**Year**	**Country**	**Study design**	**Frequent attendance definition**	**Sample**	**Main outcomes**
Garralda *et al* ^^14^^	1998	UK	Cross-sectional	≥4 attendances in 12 months	109 frequently attending children aged 7–12 years, registered with 2 practices	21% of children accounted for 58% of all attendances in 12 months.Disordered children had educational difficulties and problems in social relationships, and came from dysfunctional homes.
Vila *et al* ^^15^^	2012	UK	Cross-sectional	≥4 attendances in 12 months	1116 secondary school pupils	30% were frequent attenders.Frequent attenders were younger; more likely to come from lower socioeconomic backgrounds; reported physical problems; had more hospital visits; had more recent intense somatic symptoms, made worse by stress and causing impairment; had more days off school; had presence of emotional symptoms and a history of mental health consultations; were more likely to see a hospital doctor; and had current illness.
Fosarelli *et al* ^^19^^	1987	US	Prospective cohort	Low usage 0–4 visit/years;medium usage 5–6 visit/years;high usage ≥7 visit/years(Different thresholds were used for subsequent years)	293 infants to 3 years old attending hospital-based primary care clinic	Frequent users of one facet of care remained frequent users of other facets for all of 3 years. Children who used the clinic more frequently were more likely to have registered before 1 month of age, have multiple chronic conditions, and demonstrate consistently frequent use for health maintenance throughout the 3 years. Frequent users were also more likely to have zero or one sibling.
Stojanović-Špehar *et al^17^*	2007	Croatia	Retrospective cohort	>75 th percentile of consultation frequency	964 preschool children (1–6 years of age) from 6 family practices, and a paediatric clinic	255 were frequent attenders, with a median visit frequency of 10/year.Age of child (2–3 years), and certain symptoms and conditions (infectious, respiratory, and dermatological conditions) were associated with frequent attendance, and parents of frequent attenders were more likely to have secondary education. Those seen by a paediatrician were also more likely to be frequent attenders.
Shraim *et al* ^^16^^	2014	UK	Prospective cohort	Once in year 1, and at least once in the following year.	1437children (2–16 years) registered with primary practices contributing to a primary care database, who saw a doctor for non-specific physical symptoms in 2009	27% of children (*n* = 390) had repeated consultations for non-specific physical symptoms. Consultations for any non-specific physical symptoms in mothers was associated with increased risk of repeated consultations for non-specific physical symptoms among children.
Martin *et al* ^^18^^	2018	Spain	Cross-sectional questionnaire survey	>90th percentile/ ≥6 visits in the last 6 months	346 children (6 months–14 years) attending paediatric clinics in 2 urban primary care centres	33 high frequency users of primary care services (*n* = 55 when emergency or urgent care services included) identified.Younger age of child (<2) and higher anxiety levels in parents (GADS≥4) were related to being frequent users of primary care clinics.

GADS = Goldberg Anxiety and Depression Scale

### ​Risk of bias assessment

All six studies were found to be of fair quality (Supplementary Table 2). None provided sample size justification, power justification, variance and effect estimates, and none reported blinding of outcome assessors or loss to follow up.^14–19^ In three studies, the participation rate of eligible persons could not be determined.^14,15,17^ Two studies did not examine different levels of the exposure as related to the outcome.^14,15^

### ​Definitions and rates of frequent attendances

Garralda *et al* ^14^ defined frequent attendance as four or more attendances during a 12 month period. Twenty-one per cent of 109 frequently attending children accounted for 58% of all attendances. The authors compared frequent attenders with psychiatric problems with frequent attenders with no psychiatric problems. Vila *et al* ^15^ used the same definition, and reported that 30% of 1116 children were frequent attenders. They compared characteristics of frequent attenders with non-frequent attenders. Fosarelli *et al* ^19^ followed up for 3 years 293 children who had been enrolled in the clinic as infants. They categorised each child into a usage category: low usage (0–4 visits/year), medium usage (5–6 visits/year), and high usage (≥7 visits/year) for the first year. During the 3 years, there was a gradual reduction in the number of visits. Sixty-seven per cent attended >5 times during the first year. In the third year, 31% attended >3 times. Martin *et al* ^18^ investigated ‘*hyper-attendance*’ in a questionnaire survey of 346 children. Those who made ≥6 visits over 6 months were considered high users. They found a total of 33 children to be high users according to this definition. Stojanovic-Spehar *et al* ^17^ used a sample of preschool children, between the ages of 1–6 years. Frequent attenders were defined as those over 75^th^ percentile of consultation frequency. These were compared with non-frequent attenders, defined as those under 25^th^ percentile of consultation frequency. From a sample of 942 preschool children, 255 frequent attenders had a median number of 10 consultations (range 4–26) compared with 1 (range 0–2) in non-frequent attenders. Shraim *et al* ^16^ analysed a cohort comprised 1437 children, aged 2–16 years, who consulted a physician for non-specific physical symptoms (NSPS). They found a median number of consultations in a 2-year period was 2 (range 1–12) for children exposed to maternal consultations for NSPS compared to one consultation (range 1–16) for unexposed children.

### ​Sociodemographic and medical characteristics of children associated with frequent attendance in primary care

The sociodemographic and medical characteristics associated with frequent attendance in primary care are summarised in Table 1.

#### Sociodemographic characteristics

Garralda *et al* compared frequent attenders with psychiatric problems against frequent attenders with no psychiatric problems.^14^ More disordered children came from broken homes, and had psychosocial and health stresses.^14^ They found no significant association between psychiatric disorder in frequently attending children and parental socioeconomic status, maternal age, or education.^14^ However, Vila *et al* found that frequent attenders were significantly more likely to come from lower socioeconomic groups, be younger, and to have more school absence, relative to non-frequent attenders.^15^ Fosarelli *et al* showed that those who used the clinic frequently for health maintenance were more likely to have registered before 1 month of age. Frequent users for illness were more likely to have zero or one sibling.^19^ Shraim *et al* reported that in children exposed to maternal consultations for NSPS, more were 'not first' in birth order than in unexposed children.^16^ Martin *et al* ^18^ found that older children were less likely to be frequent attenders, with children aged <2 years making the most visits to the paediatric primary care clinics.^18^ Stojanovic-Spehar *et al* showed that, among frequent attenders, there was significantly more children aged between 2–3 years, and attending daycare centres than among non-frequent attenders. In the group of frequent attenders, significantly more parents had secondary education.^17^

#### Medical and psychosocial characteristics

Vila *et al* ^15^ showed frequent attenders were significantly more likely to report more past and current problems, more intensive physical symptoms on the children’s somatisation index, symptom-related impairment, and symptoms worsening with stress. Depressive symptoms were higher in frequent attenders, with higher total emotional and conduct scores. More frequent attenders had seen a hospital doctor for physical problems in the previous year, and more reported seeing a counsellor/psychiatrist or psychologist at some stage. Significant predictors of frequent general practice attendance included seeing a hospital doctor, reporting a current illness, having ever seen a counsellor/psychiatrist or psychologist, number of school days off school, and younger age.

Garralda *et al* ^14^ reported there was little difference in the types of presenting complaints in disordered (children with a psychiatry diagnosis who were frequent attenders) and non-disordered children (who were frequent attenders also). Disordered children were regarded by their mothers to be in poorer health. 31% of mothers of disordered children reported their child handicapped by physical problems, but only 7% for non-disordered children. Fosarelli *et al* ^19^ showed that high clinic use for illness was related to the presence of a diagnosed chronic condition. Stojanovic-Spehar *et al* ^17^ reported that frequent attendance was associate with the symptom of nasal discharge, and diagnoses of infectious and parasitic diseases, middle ear diseases, respiratory system diseases, and skin and subcutaneous tissue diseases. More frequent attenders were treated by paediatricians than by family physicians compared to non-frequent attenders. Shraim *et al* ^16^ reported children with repeated consultations commonly attended due to back pain, constipation, and abdominal pain. Overall, 27% of children had repeated consultations for NSPS. Exposure to maternal consultation for NSPS was associated with a 21% increase in consultation frequency for NSPS. Martin *et al* found children of parents with a high level of anxiety tended to attend frequently.^18^

## Discussion

### ​Summary

The articles explored the relationship between frequent attendance and sociodemographic, medical, and psychosocial characteristics. There was no consensus on the definition of frequent attenders. Various sociodemographic and medical characteristics of children were associated with frequent attendance in primary care.

### ​Strengths and limitations

To the authors' knowledge, this is the first systematic review on child frequent attendance in primary care. This study did not specify a predefined proportional threshold in terms of attendances per year or month. Additionally, some of the studies selected for inclusion sought to collect information on the sociodemographic characteristics of the participants.

This study found only six studies, despite a comprehensive search. Only observational studies were included in this review. While the eligible studies were of fair methodological quality, none provided sample size justification, variance and effect estimates, and there was no blinding of outcome assessor or reporting of whether there was loss to follow-up. Some provided little information regarding the participation rate.

Comparison between countries was challenging due to the different health system structures, cultures, and financing of service providers. Some studies relied on self-reported data, which are prone to recall bias. Due to the small number of studies meeting the eligibility criteria, publication bias was not assessed. Finally, only articles published in English were included in this review, which might have meant studies reported in non-English languages were omitted.

### ​Comparison with existing literature

The visit frequency of frequent attenders in two studies was ≥4 visits per year.^14,15^ The smallest visit frequency considered was two visits, compared with one in the comparison group over a 2-year period,^16^ but in two articles the visit frequency was much higher, at ≥6 visits per year,^18^ and ≥7 visits per year, respectively.^19^ The number of visits reported in another study was 10 per year.^17^

There is no generally accepted definition of frequent attendance.^20^ A popular approach to define top attenders is using a proportional threshold (top 25% and 10%), and stratifying by age and sex of all enlisted primary care patients.^10^ Using 10% of all enlisted primary care patients, and stratifying by age and sex is arguably the best method to select exceptional attenders, and permits comparison between practices, periods, and countries within a timeframe of 1 year.^21^ Epidemiological evidence has shown that sex- and age-adjusted prevalence rates of persistent frequent attenders are 3–5 times higher than non-frequent attenders.^21^

Previous reviews on frequent attenders have focused on adults and older people attending primary care.^3,12^ None of the studies identified reported directly on GP workload, but a previous review showed that frequent attenders take up a substantial proportion of the GP workload due to complex needs, including physical, psychological, and social problems.^22^

Results from included studies showed different proportions of child frequent attenders. This could be due to the differences in accessing services in respective countries, in focus and study research question, population, and period the study was conducted in, and the way frequent attenders were defined. The three UK studies included in this review had similar proportions of frequent attenders.

### ​Implications for research and practice

Developing alternative approaches to meet local health needs to improve care and reduce attendance among the highest users is also essential for a sustainable primary care system. Additional GPs are needed to meet the demand,^23^ but recruiting more GPs is challenging, and is only part of the solution. As frequent attenders often have a mixture of medical and psychosocial needs, person-centred and integrated care approaches can fill the gap in health provision. Aligning GP services with social care may help to highlight and manage the needs of frequently attending children, and help support families in appropriate use of GP services. Systematic reviews show the association of social isolation and child health,^24,25^ but there is limited evidence available on the appropriate clinical response in primary care. One systematic review recommended offering mothers improved access to psychological therapies and parenting programmes to mitigate the impact of maltreatment on children.^26^ Increased healthcare utilisation is associated with loneliness, which is common in primary care.^27^ Screening for child and maternal loneliness may help identify those with other social needs. Essential resources should be made available to address underlying psychosocial needs where these are identified. However, the academic community should bear in mind that many frequent attenders have predominantly medical needs stemming from ongoing medical matters with no psychosocial background, and these need enhanced clinical care.

Various sociodemographic and medical characteristics of children were associated with frequent attendance in primary care. Frequent attendance can be characterised by presence of psychosocial and mental health problems, younger age, school absence, presence of a diagnosed chronic condition, and high level of anxiety in their parents. Future research is needed to equip GPs with validated scales to screen for social connections and isolation in children and their parents or caregivers, and research on interventions needs to account for social context and community characteristics. Aligning GP services with social care may help to highlight and manage the needs of frequently attending children, and help support families in appropriate use of GP services.
